# A retrospective study of ophthalmologic presentation, management, and outcomes in pediatric patients admitted with abusive head trauma

**DOI:** 10.3389/fmed.2024.1416626

**Published:** 2024-08-15

**Authors:** Jiawei Yin, Jie Peng, Xuerui Zhang, Yuan Yang, Victoria Y. Gu, Wenting Zhang, Huanyu Liu, Haodong Xiao, Yu Xu, Peiquan Zhao

**Affiliations:** ^1^Department of Ophthalmology, Xinhua Hospital Affiliated to Shanghai Jiao Tong University School of Medicine, Shanghai, China; ^2^Shanghai Jiao Tong University School of Medicine, Shanghai, China; ^3^Johns Hopkins Bloomberg School of Public Health, Baltimore, MD, United States

**Keywords:** abusive head trauma, ocular manifestations, retinal hemorrhage, peripheral ischemic retina, ophthalmological assessment

## Abstract

**Background:**

Abusive head trauma (AHT) is a severe form of physical abuse leading to significant morbidity and mortality in children, often presenting with complex brain injuries. Among the varied manifestations, ophthalmologic presentations are critical yet underexplored, which may provide essential clues for early diagnosis and management, improving long-term visual and neurological outcomes.

**Objective:**

This study aims to explore the manifestation, management, and outcomes of AHT cases within a single center in China over a five-year period, with a focus on the importance of ophthalmologic evaluation in enhancing the diagnosis, management, and outcome predictions of AHT.

**Methods:**

A retrospective case series was conducted at a single institution, involving infants diagnosed with AHT from 2019 to 2023. Data on demographics, medical histories, and clinical management were collected. Ophthalmologic examinations including fundus photography, ocular B-scan ultrasound and fundus fluorescein angiography (FFA), were performed to evaluate retinal vasculature and identify peripheral ischemic retina (PIR). Statistical analyses were performed using SPSS ver. 26.0.

**Results:**

Eight AHT patients (16 eyes) were included in the study. Bilateral ocular involvement was observed in all patients, with 81.25% exhibiting retinal hemorrhages (RH). Other manifestations included retinal detachment (31.25%) and optic nerve atrophy (18.75%). Clinical interventions varied, with 68.75% of patients undergoing treatments such as laser photocoagulation and anti-vascular endothelial growth factor (VEGF) injections. Among all eyes, 75% showed resolution of RH. Despite treatment, some patients progressed to severe conditions such as retinal detachment (RD) and iris neovascularization (INV).

**Conclusion:**

This study emphasizes the importance of a multidisciplinary approach in the diagnosis and management of AHT, particularly by integrating ophthalmological perspectives into patient care. These findings contribute to the understanding of ophthalmologic presentations in AHT.

## 1 Introduction

Abusive head trauma (AHT), the primary cause of death and traumatic cranial injuries among infants, refers to injuries caused by physical abuse to the brain or skull. Globally, the estimated incidence rate is approximately 35 per 100,000 infants under the age of one, with noticeable mortality rates (1–4). AHT is the most prevalent among infants aged 2–4 months when continuous infant crying peaks, exacerbating the stress of caregivers (1, 5). Key risk factors frequently associated with caregivers include poverty, single parenthood, insufficient parental awareness, and unstable family situations (6). Survivors would suffer lifelong impairments, such as blindness, developmental problems, and neurological sequelae, which results in long-term costs for medical treatment, daily-life assistance, and increased financial burdens.

Identifying symptoms of AHT, including cerebral edema, ocular hemorrhages, neurological disorders, and occasionally bone fractures, can be challenging due to their nonspecific manifestations and often the absence of a definitive traumatic history. Patients usually manifest reduced social activities, decreased concentration, eating disorders, drowsiness, and poor growth in the early stages of the disease (7). The diagnosis of AHT can be complicated and often delayed, which causes the need for additional diagnostic procedures including more detailed imaging and ophthalmological evaluations. Ocular manifestations are critical yet underexplored aspects of AHT, providing essential clues for early diagnosis and management, which improve long-term visual and neurological outcomes. AHT often presents with a spectrum of ocular findings, with RHs being the most commonly reported ocular manifestation. Previous studies have shown that the incidence of RH can exceed 80%, significantly higher in AHT patients than in those with accidental head injuries (8–11). Moreover, RH has been found to have a high sensitivity for diagnosing AHT (12). Therefore, RH is an essential indicator in both the identification and treatment of AHT, requiring prompt assessment (1, 13–15). Other ocular findings include perimacular folds, retinoschisis, optic nerve sheath hemorrhages, and less commonly, vitreous hemorrhages, which help differentiate AHT from other causes of RH. The potential ocular complications arising from AHT are severe and can lead to long-term visual impairment. Persistent retinal damage can result in conditions such as amblyopia, RD, and optic atrophy (12, 16, 17). Although a considerable proportion of AHT patients present improvements including reattached retina after clinical interventions, some AHT patients suffer from poor visual prognosis and long-term visual impairment, which are not evident upon hospital discharge (16, 18, 19).

To address the research gap in the specific ocular injuries of pediatric AHT patients in China, a multidisciplinary diagnostic approach was employed in this study to describe the ocular manifestations seen in pediatric AHT patients from a single center. By presenting the ocular manifestations and their association with AHT, the research aims to identify discrepancies with previous studies, which thereby contributed valuable insights into the identification and management of AHT.

## 2 Method

### 2.1 Study design and participant recruitment

The study is a retrospective, consecutive, single-institutional case series that included infants diagnosed with AHT from 2019 to 2023 at the Xinhua Hospital affiliated with Shanghai Jiao Tong University School of Medicine. It gained the approval from the Ethics Committee of the hospital (No. XHEC-D-2024-067) and abided by the tenets of the Declaration of Helsinki. The participation of patients and the use of their medical records and images were executed following the acquisition of informed consent from their legal guardians. After the initial consultations in various departments, patients who developed ocular symptoms were referred by specialists to the ophthalmology department, including pediatricians, emergency department physicians, and neurologists. The inclusion criteria for the diagnosis of AHT were established through the traditional triad, including the concurrent presence of subdural hemorrhage (SDH), RH, and brain dysfunction through multi-disciplinary consultation, and based on the history of physical abuse by caregivers or specific injury scenarios (2, 7). Cases of RH caused by other disorders including retinopathy of prematurity and familial exudative vitreoretinopathy, individuals lost to follow up and those without complete medical records were excluded from the study to ensure a focused study cohort.

### 2.2 Ophthalmologic examinations and outcome measures

Demographic information, detailed medical histories, and clinical management approaches, including laser photocoagulation (LP), anti-VEGF injection, lensectomy, and pars plana vitrectomy (PPV) were documented. Outcome measures included ocular manifestations in the affected eyes, clinical interventions provided, complications, and final anatomical states in the affected eyes. The need for further treatment relied on follow-up fundus findings. Fundus findings were recorded using Retcam III fundus photography (Clarity Medical Systems, California, United States). A pale optic disc was defined as optic nerve atrophy (ONA). Ocular B-scan ultrasound was performed using Aviso B-scan (Quantel Medical, France), and indirect ophthalmoscopy were used to identify the site of ocular bleeding and concomitant RD. The anterior segment of the eye was examined using the RetCam system. Images of the anterior segment were captured to assess the presence of traumatic cataracts and other anterior segment abnormalities. FFA was performed using the RetCam system to evaluate the retinal vasculature and identify vascular abnormalities or leakage. RHs were classified into three categories: mild, moderate, and severe. Mild RHs are characterized by intraretinal hemorrhages only. Moderate RHs include lesions smaller than two-disc areas. Severe RHs are marked by lesions exceeding two-disc areas in size (20). For patients initially referred to the ophthalmology department with AHT, follow-up was conducted within 1–2 weeks to assess hemorrhage absorption. Patients were scheduled for a follow-up appointment at 4 weeks postoperatively to assess early outcomes, and subsequent follow-ups were arranged between 3 and 6 months to monitor outcomes and any complications. Anatomical improvement was defined as the resolution of RHs, the negation of the need for additional interventions, or a stable situation across the final two follow-up visits.

### 2.3 Statistical analysis

Medical information was collected from medical records and documented in a Microsoft Excel spreadsheet. Quantitative data were represented as median values, and qualitative data were described as the actual count and relative distribution. Various statistical tests, including chi-square or Fisher’s exact tests, were performed to analyze the level of significance. A *p*-value less than 0.05 was considered statistically significant. The data were analyzed using the Statistical Package for the Social Sciences [SPSS ver. 26.0, Chicago, Illinois (IL), United States].

## 3 Results

### 3.1 Characteristics and presentation of patients

Eight patients with AHT were included in this study, contributing to a total of 16 eyes in the analysis. Table 1 lists the baseline demographic and clinical presentations. Four patients (50%) were under 1 year old, and the average age of all patients at the first visit was 15.13 ± 11.98 months (range, 4–41 months). Follow-up duration varied significantly among patients, ranging from 1 to 15 months. The mean follow-up duration of all individuals was 8 ± 5.88 months. At presentation, all patients had bilateral eye involvement. They exhibited neurological disorders at the first ophthalmologic visit. The cohort consisted of 75% males (six out of eight) and 25% females (two out of eight). Preterm birth was identified in 25% of the cases (two out of eight). Both presented with bilateral ocular involvement, which was similar to the rest of the cohort. Notably, 75% of the cases had an explicit history of trauma, with initial presentations predominantly managed by neurosurgeons or emergency physicians, which indicated the severity of their conditions. Extracranial manifestations were diverse. Among them, SDHs were the most common, followed by various forms of hemorrhage and brain injuries, highlighting the complexity and severity of the traumas encountered. No death occurred.

**Table 1:** Demographic, manifestations, management and outcomes.

Patient	Age at the first visit (months)	Age at the first treatment, months	Follow-up time	Gender	Preterm infant	Ocular involvement	Explicit history of trauma	Primary specialist consultant	Extraocular manifestations	Eye (no. of eyes)	Initial ophthalmic presentation	Management	Final outcomes
1	17	18	13	F	No	Bilateral	Yes	Neurosurgeon	Cerebral oedema, seizure	OD	—	—	ONA, RH resolution
										OS	Mild RH, temporal PRH, RE, PIR	IV + LP	ONA, ERM, RH resolution
2	16	17	8	M	No	Bilateral	Yes	Emergency physician	Subdural hemorrhage	OD	Massive RH, RD, PIR	IV + LP	RF, ERM, SRE, RH resolution
										OS	Massive RH, RD, PVR, RE, PIR	IV + LP, PPV + ERM peeling + SO (secondary treatment)	Total RD, INV, RH resolution
3	11	12	1	M	No	Bilateral	Yes	Emergency physician	Brain atrophy, Intracranial hemorrhage	OD	Mild RH, PRH, PIR	IV + LP	Persistent RH
										OS	Massive RH, PRH, PIR	IV + LP	Persistent RH
4	6	—	13	M	No	Bilateral	Yes	Neurosurgeon	Subdural hemorrhage	OD	PIR	—	RH resolution
										OS	Moderate RH	—	ERM, RPE
5	4	8	11	M	Yes	Bilateral	Unknown	Pediatrician	Intracerebral hemorrhage	OD	Moderate RH, RE, PVR, RD, PIR	IV, L + PPV + ERM peeling + gas + OVD (secondary treatment), SR (third treatment)	Total RD, INV, RH resolution
										OS	Moderate RH, RE, PVR, RD, PIR	IV	Total RD, RH resolution
6	6	7	1	F	Yes	Bilateral	Yes	Neurosurgeon	Seizure, subdural hemorrhage, cerebral contusion	OD	Massive RH, MH, SRH, PIR	LP + PPV + ERM peeling + SO	RH resolution
										OS	Massive RH, PIR	LP + PPV + Gas	RH resolution
7	41	—	2	M	No	Bilateral	Yes	Orthopedic surgeon	Subdural hemorrhage, intracranial hypertension, brain atrophy, long bone fractures, orbital fracture	OD	Massive RH	—	ONA, persistent RH
										OS	Massive RH, PVR, RD, traumatic optic neuropathy	LP + PPV	ONA, RD, SRF, RH resolution
8	20	30	15	M	No	Bilateral	Yes	Emergency physician	Skull fractures, long bone fractures	OD	PIR	—	ONA, RH resolution
										OS	Massive RH, SRH, PIR	PPV + ERM peeling + Gas	ERM, RH resolution

RH, retinal hemorrhage; SRH, subretinal hemorrhage; MH, macular hole; RE, retinal exudation; PIR, peripheral ischemic retina; PVR, proliferative vitreoretinopathy; PRH, preretinal hemorrhage; RD, retinal detachment; IV, intravitreal injection; LP, laser photocoagulation; PPV, pars plana vitrectomy; ERM, epiretinal membrane; SO, silicone oil; SR, subretinal injection; L, lensectomy; OVD, ophthalmic viscosurgical device; ONA, optic nerve atrophy; RF, retinal fold; SRE, subretinal retinal exudation; INV, iris neovascularization; SRF, subretinal fluid.

### 3.2 Ocular manifestations and clinical management of patients

Abnormal ocular manifestations were present in 15 of 16 eyes (93.75%), and all patients had poor focusing and tracking abilities. RH, the most common symptom, was present in 13 of 16 eyes (81.25%). RD was observed in five eyes (31.25%). Proliferative vitreoretinopathy was found in four eyes (25%). Retinal exudation was detected in four eyes (25%). A macular hole was observed in one eye (6.25%), which also presented with PIR, and subretinal and massive RHs (Table 1). Through binocular ophthalmoscopy, two patients exhibited unilateral persistent RHs. Among the patients, three had ONA at the follow-up visit. None of the patients developed traumatic cataracts.

Of the 16 eyes evaluated, five (31.25%) did not undergo any clinical interventions, while 11 (68.75%) underwent various kinds of clinical management. All eyes that did not receive ophthalmologic management exhibited anatomical improvements. The median age of patients at the first treatment was 15.33 ± 8.48 months (range, 7–30 months). Six out of 16 eyes (37.5%) underwent PPV during follow-up due to proliferative vitreoretinopathy or RD.

### 3.3 Indications and timing for clinical management

Due to the lack of significant improvement, indicated by persistent hemorrhage or proliferative changes during follow-up, most of AHT cases (62.5%) underwent FFA and presented with PIR. The criteria for laser treatment in the research were based on the severity of the ischemia and the presence of progressive retinal changes. For cases with significant PIR detected by FFA, laser treatment was utilized. Two eyes with PIR did not have RHs, and no treatment measures were taken for these eyes. Five eyes (31.25%) underwent LP and/or anti-VEGF injection without further treatment. RHs were present unilaterally or bilaterally in each patient, with some cases also presenting RD. In cases of disease progression during follow-up, additional treatments were administered based on individual patient conditions. Four treated eyes developed more serious conditions, such as RD or iris neovascularization (INV), all of which had PIR. Two of these eyes underwent additional treatment. Among all eyes, 12 (75%) experienced resolution of RH, and five exhibited ONA during the follow-up period. Ultimately, five eyes (31.25%) showed improvement throughout the follow-up period.

**Figure 1:**
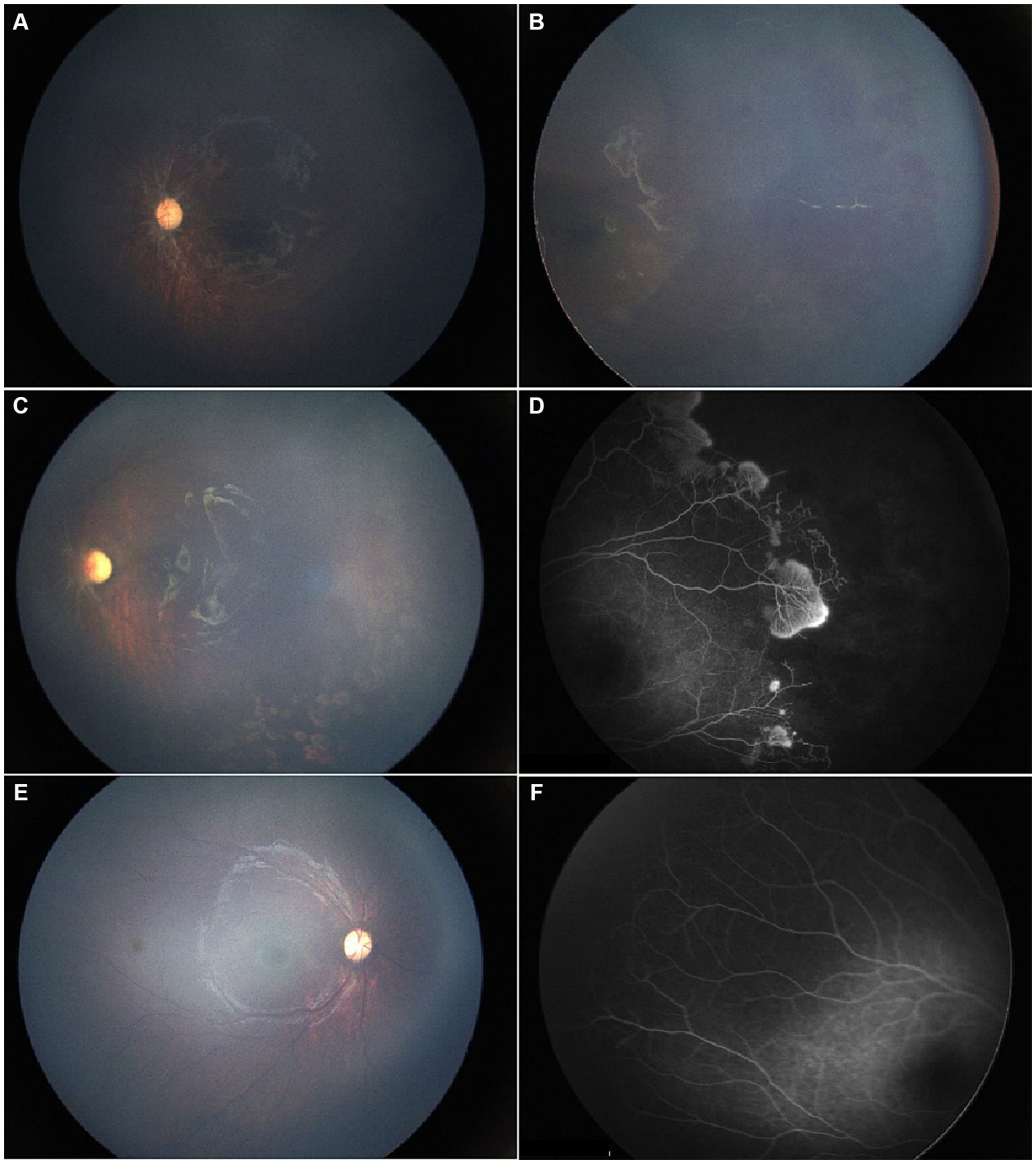
A 17-month-old female patient presented for the first time with a history of hydrocephalus, impaired vision, and headache. **(A,B)** Initial evaluation showed diffuse RH in the temporal region of the left eye, vitreous opacity, and dragged-disc-like retinal traction. **(C)** Following unilateral laser photocoagulation and anti-VEGF therapy, resolution of vitreous hemorrhage with no recurrence was observed at the one-month follow-up, consistent with improved visual function. **(D)** FFA revealed significant peripheral avascular retina and leaking blood vessels in the left eye. **(E,F)** By the last visit, the right eye was under clinical observation with a normal fundus presentation, while the optic disc was pale. Meanwhile, the retinal traction in the left eye with minor proliferative membranes remained stable.

**Figure 2:**
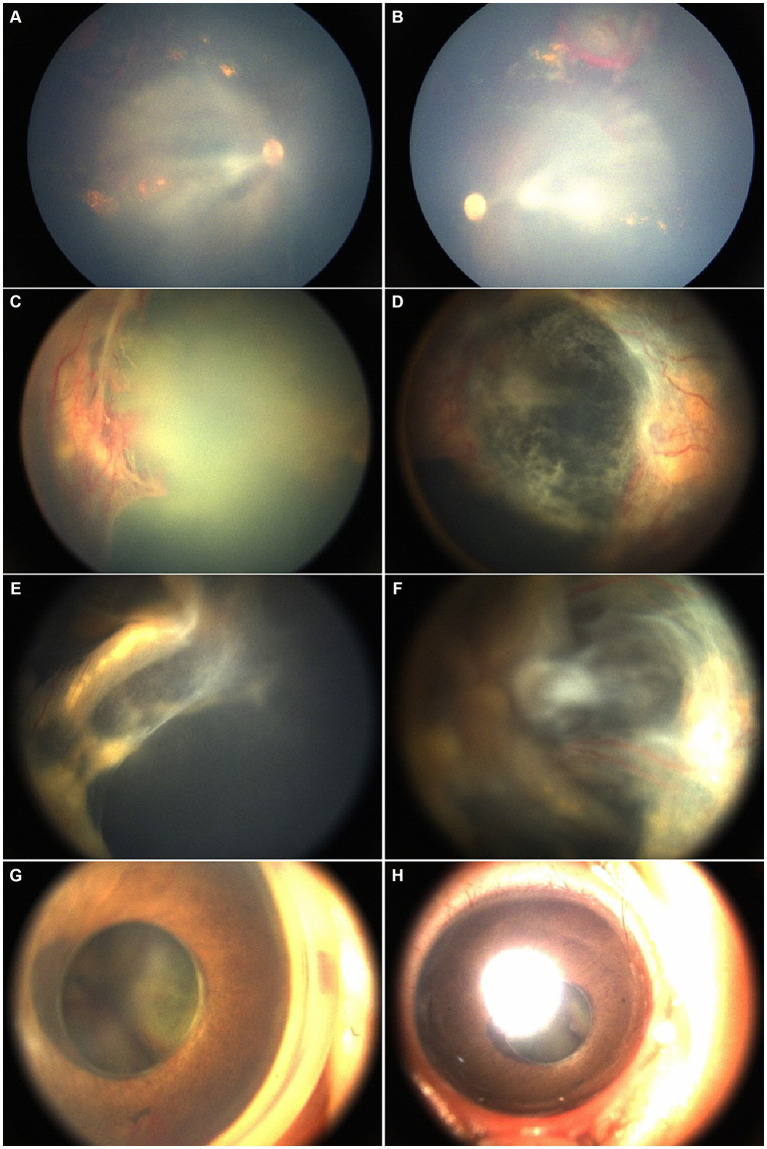
A male patient was 4 months old at the time of his first consultation. His past medical history included resolving intracranial hemorrhage with secondary ocular complications, including bilateral chronic and recurrent RH, fundus exudation, and proliferative membranes. Initial fundus examination under poor visualization of the posterior suggested predominantly flat retinas. Rapid progression to bilateral retinal detachment, RH, and tortuous vessels was noted at the second visit. An anti-VEGF injection was administered followed by right eye lensectomy and PPV resulting in partial retinal reattachment. However, 3 months later, at the subsequent visit, INV developed in the operated eye necessitating subretinal injection of anti-VEGF. **(A,B)** Fundus images from the first visit. **(C,D)** After 3 months, preoperative fundus images from the second visit show the rapid progression of retinal progression and tortuous vessels, indicating the need for further treatment. **(E,F)** Fundus images postoperatively. **(G,H)** The anterior segment images, 4 months after second treatment depict iris neovascularization with funnel-like retinal detachment in the right eye after the subretinal anti-VEGF injection.

**Figure 3:**
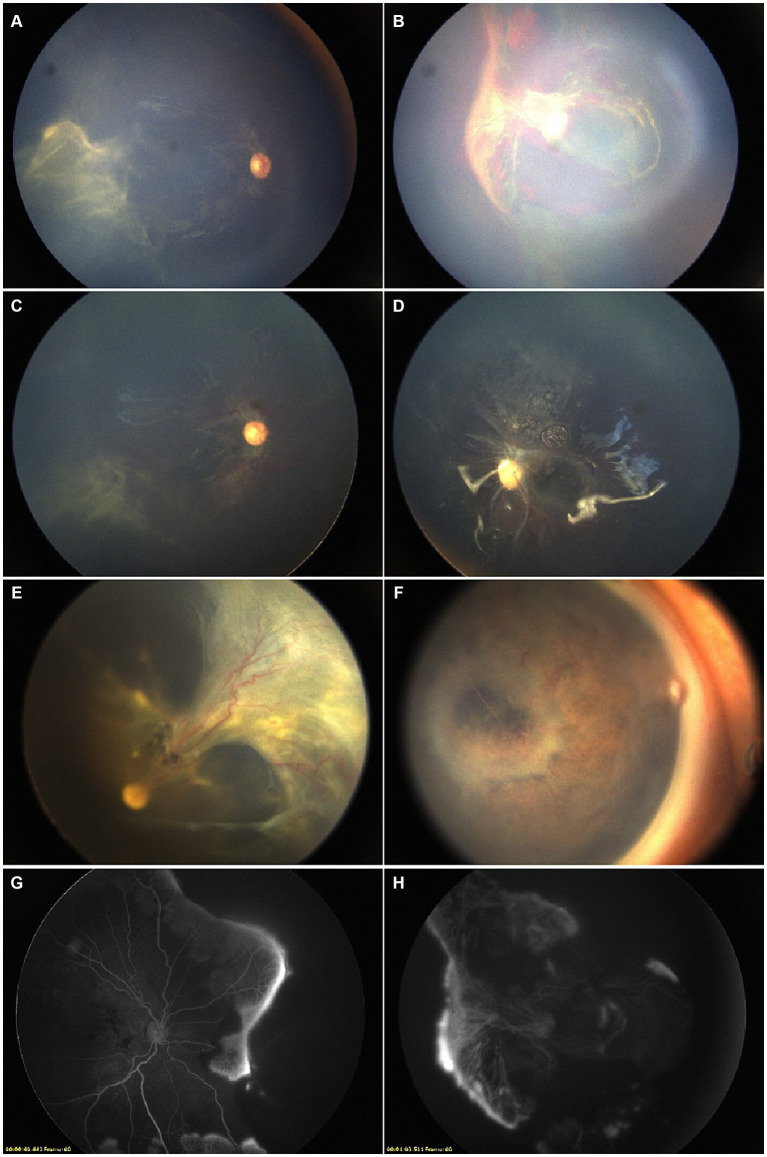
A male patient was 16 months old at his first consultation. The diagnosis of AHT was established based on a history of brain injuries and cephalic symptoms. **(A,B)** Fundus images prior to interventions. Bilateral laser photocoagulation and intravitreal anti-VEGF injections were performed, resulting in partial remission. **(C,D)** Pre-surgical FFA showed partial retinal vasodilatation and fluorescein leakage in the peripheral circulatory detached retina. Fundus images presented macular obscuration due to RH. The patient underwent PPV, removal of proliferative membranes, and silicone oil tamponade. **(E,F)** Monthly postoperative assessments showed stabilization, with the resolution of the diffuse RH and improved peripheral retinal detachment. **(G,H)** However, post-PPV fundus images showed the formation of retinal fold in the right eye, and the presence of INV in the left eye during the final visit.

Initial examinations revealed that one patient’s retina was generally flat with PIR. Due to geographical and socioeconomic factors, the patient was unable to return for timely follow-up and FFA examination. The patient exhibited rapid progression to bilateral RD, accompanied by dispersed intraretinal hemorrhage and tortuous vessels at the second visit 3 months later. An intravitreal anti-VEGF injection was administered. Simultaneously, to resolve the shallow anterior chamber, a lensectomy and PPV were performed on the right eye, resulting in partial unfolding of the retina postoperatively. At a follow-up visit 3 months later, INV developed in the postoperative eye. Subsequently, a subretinal injection of an anti-VEGF drug was administered, effectively reducing the pathological angiogenesis on the iris. Examples of ocular manifestations associated with AHT are shown in Figures 1–3.

## 4 Discussion

This study is the first to report the ophthalmologic manifestations, management, and outcomes of Chinese AHT patients. It was found that all patients with AHT manifested ophthalmologic symptoms, and most were under the age of three. The rapid development of RD and INV seen in a single AHT case following subretinal anti-VEGF injection in this study was not reported in previous studies. The analysis of this study revealed the critical role of FFA in identifying PIR, which influenced the choice of subsequent treatments including LP and intravitreal (IV) injection. This highlights the potential role of these interventions in reducing further complications.

A retrospective analysis was performed on the ophthalmologic outcomes of AHT patients attending follow-up appointments. The study findings indicate that all AHT patients were first presented to medical specialties other than ophthalmology. SDHs are the most common extraocular injury in AHT patients (21). Based on previous studies, the incidence rate of SDHs varies from 46 to 93% (1, 9, 18). Similarly, this study found that 50% of cases presented SDHs, observed as the predominant extraocular injury. Additionally, a high incidence of SDHs was not correlated with mortality. Nevertheless, cases of this study were reported to suffer persistent neurological impairments such as seizures, motor disabilities, and cognitive deficits. These abnormalities are likely associated with the lasting impact of intracranial damage in AHT patients due to the vulnerability of infants to physical factors (1).

RH is the most commonly reported ocular manifestation related to AHT, with an incidence rate of 5.3 to 60% (15, 22). Pierre-Kahn et al. (11) found that intraocular hemorrhages were a more prevalent symptom in infants without skull involvement, including shaken baby syndrome. In contrast, research on AHT presented a lower incidence rate of RH. Weiss et al. (15) reported a less frequent occurrence of RHs at 5.3%. In this study, however, a much higher incidence of RH (81.25%) was found. This difference may be due to the limited number of participants and the majority of patients referred from other departments.

RH alone is not an exclusive indicator of AHT, as it may also result from other diseases. Unlike AHT, RH due to increased intracranial pressure is rare and typically presents as intraretinal hemorrhages located adjacent to a swollen optic disc (23). Coagulopathies, including clotting factor deficiencies and disseminated intravascular coagulation, can also cause RH, although these conditions typically lack the severity and distinct characteristics observed in AHT. In critically ill children, severe hemorrhagic retinopathy is rare and generally associated with other diagnoses. Premature infants, especially those with retinopathy of prematurity (ROP), may exhibit RH, which does not necessarily indicate non-accidental injury (24, 25). Therefore, additional evidence is needed for diagnosis. Previous studies identified a controversial association between SDH and RH, which suggested that the concurrence may strongly imply a nonaccidental shaking behavior (11). In the present study, no significant correlation was detected between SDH and RH (Supplementary Table S1). In China, many parents lack a comprehensive understanding of AHT and its subtle symptoms, leading to a failure to seek medical care promptly (26, 27). Additionally, one-third of AHT patients were visually alert at their first medical encounter. Being retrospective, this study may have overlooked ocular manifestations of previous AHT cases due to delayed fundus examinations or untimely referrals to ophthalmology (8). This oversight might also explain the smaller sample size.

The precise pathogenesis of ocular lesions in AHT provokes ongoing debates. The mechanisms may be complex in most cases. In specific situations, external forces like shaking are typically linked to bilateral RHs, possibly suggesting the presence of vitreous traction (15). RD in AHT patients is typically classified as traumatic (11). This study observed a case of rapid progression of RD, which may be influenced by systemic effects of traumatic brain injury (TBI). Literature suggests that TBI can lead to systemic inflammatory responses and coagulopathies, potentially causing ischemia (28). Caputo et al. (29) reported three AHT cases with tractional RD and illustrated the potential role of retinal ischemia in ocular abnormalities related to cranial injuries. The vasculature of infants with immature nervous and vascular systems tends to be comparatively fragile and more easily disturbed. Based on Caputo’s et al. (29) study, it is suggested that the delicate vascular architecture in infants may explain their high incidence of hemorrhages. However, healthy children involved in accidental injuries may present RH in a few cases. RHs associated with accidental injuries are inclined to be mild and unilateral based on previous studies (30). While it is hypothesized that brain injuries could contribute to peripheral retinal vessel occlusion through systemic inflammatory responses and coagulopathies, further investigation is required, acknowledging that additional data and a control group are necessary to substantiate this hypothesis (29, 31, 32).

Although hypoxia-ischemic injuries caused by AHT have not been proven to lead to clinical ophthalmologic presentations, AHT-associated retinal ischemia may result in RD and preretinal fibrovascular proliferation (33). In previous studies, AHT patients with obvious ophthalmologic manifestations tended to remain in a relatively stable anatomical situations like retinal reattachment after receiving clinical management (13, 16, 19, 29). Moreover, nonperfusion retinal changes and ONA could be linked to unfavorable functional outcomes (34, 35). However, only 75% of the patients had a relatively flat postoperative retina at the last visit in this study, and two of the cases with PIR progressed to total RD even after clinical management. One case (Figure 2), initially stable after LP, deteriorated after surgery. Another case (Figure 3), who failed to visit as advised and lacked timely laser treatment, developed bilateral RD, suggesting that brain injuries may contribute to peripheral retinal vessel occlusion and rapid AHT progression. Initial examinations, including B-scan ultrasound and fundus examination, in this patient with rapidly progressing RD revealed no significant RD, suggesting that the rapid progression at the three-month follow-up could be due to PIR rather than an underlying mild RD. Among patients with PIR who received laser treatment, most remained stable, though some experienced disease progression. Based on this study’s findings, an association between PIR and the progression of postoperative conditions is suggested, differing from previous research. Further data are needed to validate this hypothesis (29, 34).

LP has been shown to effectively mitigate aberrant angiogenesis and RD (36, 37). In the study by Goldenberg et al. (38), seven out of 10 patients underwent LP, and none of the patients developed neovascularization. In this study, 43.75% of the eyes exhibited retinal ischemia, but only 71.43% underwent targeted LP. Furthermore, cerebral anoxia and ischemia may increase intracranial VEGF levels and neovascular proliferation (29, 39–43). Studies have been conducted on combined laser and anti-VEGF therapy as initial approaches to eradicate RH with improved visual outcomes in a variety of intraocular diseases. Given this, it is suggested that employing the combined treatment in AHT-associated RHs may yield positive outcomes (44–49). In this study, 71.4% of patients who underwent IV anti-VEGF treatment showed RH resolution. Although LP may stabilize the condition, continuous monitoring is essential. The small sample size limits the generalizability of these findings, necessitating further research with larger cohorts.

This study is constrained by several limitations. The relatively small sample size led to a higher standard deviation. The retrospective nature and limited follow-up duration also constrained the conclusions of this study. The present study was limited by the lack of data on focusing and tracking abilities, and visual outcomes, which would have helped better understand the causes of PIR in these patients. While this study lacked a control group for the identification of peripheral retinas, Foos and Kopelow found perfused peripheral retinas in normal age-matched children (50). In this study, a standard treatment protocol was not included. All of these variables could influence the final result.

In conclusion, this study highlights the importance of early and detailed ophthalmologic evaluations in pediatric AHT. The use of FFA to detect PIR may significantly guide treatment decisions, leading to better outcomes. Despite limitations such as small sample size and retrospective design, these findings highlight the necessity for multidisciplinary approaches and early intervention to improve long-term visual outcomes in AHT patients.

## Data Availability

The raw data supporting the conclusions of this article will be made available by the authors, without undue reservation.
