# Supplementary material for: A retrospective study of ophthalmologic presentation, management, and outcomes in pediatric patients admitted with abusive head trauma
Source: Front Med (Lausanne). 2024 Aug 15;11:1416626. doi: 10.3389/fmed.2024.1416626 (PMC11357973; doi:10.3389/fmed.2024.1416626)
Supplement: Supplementary file 1 [file Table_1.DOCX]

Supplementary Table 1. Association between subdural hemorrhage and retinal hemorrhage

|  | With retinal hemorrhage | Without retinal hemorrhage | *P* |
| --- | --- | --- | --- |
| With subdural hemorrhage | 7 | 1 |  |
| Without subdural hemorrhage | 6 | 2 |  |
| Total | 13 | 3 | .5218 |
